# Afternoon Nap and Bright Light Exposure Improve Cognitive Flexibility Post Lunch

**DOI:** 10.1371/journal.pone.0125359

**Published:** 2015-05-27

**Authors:** Hichem Slama, Gaétane Deliens, Rémy Schmitz, Philippe Peigneux, Rachel Leproult

**Affiliations:** 1 UR2NF - Neuropsychology and Functional Neuroimaging Research Group at CRCN -Center for Research in Cognition and Neurosciences and UNI - ULB Neurosciences Institute, Université Libre de Bruxelles (ULB), Brussels, Belgium; 2 UNESCOG - Research Unit in Cognitive Neurosciences at CRCN - Center for Research in Cognition and Neurosciences and UNI - ULB Neurosciences Institute, Université Libre de Bruxelles (ULB), Brussels, Belgium; 3 CO3 - Consciousness, Cognition & Computation Group at CRCN - Center for Research in Cognition and Neurosciences and UNI - ULB Neurosciences Institute, Université Libre de Bruxelles (ULB), Brussels, Belgium; 4 LABNIC - Laboratory for Neurology and Imaging of Cognition, Department of Neuroscience and Clinic of Neurology, Campus Biotech, University of Geneva, Geneva, Switzerland; University of Oxford, UNITED KINGDOM

## Abstract

Beneficial effects of napping or bright light exposure on cognitive performance have been reported in participants exposed to sleep loss. Nonetheless, few studies investigated the effect of these potential countermeasures against the temporary drop in performance observed in mid-afternoon, and even less so on cognitive flexibility, a crucial component of executive functions. This study investigated the impact of either an afternoon nap or bright light exposure on post-prandial alterations in task switching performance in well-rested participants. Twenty-five healthy adults participated in two randomized experimental conditions, either wake versus nap (n=15), or bright light versus placebo (n=10). Participants were tested on a switching task three times (morning, post-lunch and late afternoon sessions). The interventions occurred prior to the post-lunch session. In the nap/wake condition, participants either stayed awake watching a 30-minute documentary or had the opportunity to take a nap for 30 minutes. In the bright light/placebo condition, participants watched a documentary under either bright blue light or dim orange light (placebo) for 30 minutes. The switch cost estimates cognitive flexibility and measures task-switching efficiency. Increased switch cost scores indicate higher difficulties to switch between tasks. In both control conditions (wake or placebo), accuracy switch-cost score increased post lunch. Both interventions (nap or bright light) elicited a decrease in accuracy switch-cost score post lunch, which was associated with diminished fatigue and decreased variability in vigilance. Additionally, there was a trend for a post-lunch benefit of bright light with a decreased latency switch-cost score. In the nap group, improvements in accuracy switch-cost score were associated with more NREM sleep stage N1. Thus, exposure to bright light during the post-lunch dip, a countermeasure easily applicable in daily life, results in similar beneficial effects as a short nap on performance in the cognitive flexibility domain with possible additional benefits on latency switch-cost scores.

## Introduction

Vehicle accidents caused by sleepiness occur mainly between 2am and 6am but also between 2pm and 4pm [1,2]. Similarly, productivity at work exhibits a decrease during nighttime but also shortly after noon [3]. The temporary drop in alertness and performance during the afternoon, referred to as the post-lunch dip, was evidenced in laboratory studies using various cognitive tests [4–7]. The post-lunch dip is not completely explained by meal ingestion [8–11], but rather reflects the 12-hour harmonic of the circadian clock [6].

Taking an afternoon nap is an efficient countermeasure to overcome the post-lunch dip in alertness and performance. Indeed, napping 15 to 45 minutes before (i.e. around 12:30) or during the post-lunch period (i.e. around 14:00) decreases subjective sleepiness [1,12–16]. Restorative effects of a post-lunch nap on subjective daytime alertness are also observed on the worksite [17]. However, laboratory studies using more objective performance measurements report mixed results depending on the cognitive task, the timings of the nap and/or the testing, and individuals’ habit of napping or not in the afternoon (for a review see [18]). A long nap appears to be less effective to tackle a loss in alertness and a decline in cognitive functions, probably because of the resulting sleep inertia [16]. Finally, the restorative effect of napping depends on the nature of the mental work [19].

Besides napping, bright light exposure is another possible strategy to counteract the decreases in alertness and performance, especially using blue light [20–24]. Bright light is well known to benefit seasonal affective disorders [25] and to phase-shift the circadian rhythms [26], but also to improve alertness and performance on several cognitive tasks [24,27–31] (for a review see [32]). The effect of bright light exposure on cognitive performance and alertness depend on the preceding night duration, the time of day when bright light is administered and the type of cognitive tasks. Prior studies have investigated the effect of bright light exposure in the late evening (e.g. during simulated night shift work [33]), in the early morning [34], during a period of extended wakefulness including a night of total sleep deprivation [35,36], in the morning [37,38] or in the afternoon [24] following sleep restriction. Potential effects of bright light exposure have also been assessed during the daytime [24,38]. Functional magnetic resonance imaging studies have evidenced that daytime blue light exposure, as compared to green [39,40] or violet [40] light, is more effective in enhancing brain responses during a working memory task without improving behavioral performance. However, very few studies investigated the effects of bright light exposure during the post-lunch dip [41,42], which is the focus of the present research. Kaida et al. [41] showed that alpha power density during eyes open decreases upon exposure to natural bright light during the post-lunch dip, indicating a higher arousal level, without affecting performance on a vigilance task.

The beneficial effects of a post-prandial nap or bright light exposure on cognitive flexibility have been narrowly studied. In this respect, investigating how task-switching abilities are affected is particularly relevant, as switching between multiple activities is an index of cognitive flexibility, which is part of our everyday life. Task switching belongs to a broader domain of executive functioning, defined as the ability to organize and engage in goal-directed behavior [43]. However, switching from one task to another involves higher cognitive control, reflected by longer response times and more errors, as compared to repeating the same task [44]. The detrimental consequences of switching from one task to another are coined under the term of switch cost. Task switching thus provides a laboratory tool to study cognitive flexibility and control of goal-directed behavior in situations involving multiple tasks [45]. Additionally, the analysis of reactive and proactive components of task switching [46,47] allows differentiating early selection and late correction mechanisms in cognitive control. Since the seminal articles of Rogers and Monsell [47] and Meiran [48], the time interval given to participants to prepare for the next trial has been manipulated in task-switching procedures. This preparation time has been shown to influence both task reconfiguration and interference from the previous trial [45]. In agreement with this impact of preparation time, the dual model of cognitive control postulates that cognitive control operates via two distinct operating modes, ‘proactive control’ and ‘reactive control’. The proactive control can be conceptualized as a form of ‘early selection’ in which goal-relevant information is actively maintained in a sustained manner, before the occurrence of cognitively demanding events. This proactive control biases attention, perception and action systems depending on the goal. By contrast, in reactive control, attention is recruited as a ‘late correction’ mechanism that is mobilized only as needed. Thus, proactive control depends on the anticipation and prevention of interference before it occurs, whereas reactive control relies upon the detection and resolution of interference after its onset [46]. Proactive control reflects the sustained and anticipatory maintenance of goal-relevant information within the lateral prefrontal cortex (PFC) to enable optimal cognitive performance, whereas reactive control reflects transient stimulus-driven goal reactivation that recruits a wider brain network based on interference demands or episodic associations [46]. Measuring both proactive and reactive components of task switching is of particular importance when investigating the potential effect of an intervention.

The impact of sleep on task switching has been studied in the context of sleep deprivation [49,50], chronic sleep restriction [51], circadian variations [52,53], sleep inertia [54] and sleep disorders like insomnia [55]. These studies disclosed negative consequences on task switching, characterized by an increased switch cost, when sleep is disturbed, and a modulation of switch cost by sleep homeostatic and circadian variations. To the best of our knowledge, no study has investigated the impact of a nap or bright light exposure on task switching during the post-lunch dip under habitual sleep conditions. In a recent study, napping for 20 minutes was found to benefit performance on task switching, but not bright light exposure [42]. However, participants reduced their normal sleep by about 20% before the intervention. *Ad libitum* naps taken by interns during a night shift improved global performance speed for both repeat and switch trials during task switching in the morning without specifically affecting the switch cost when compared to interns that remained awake [56]. However, reduction in habitual sleep amounts in these two latter studies prevents drawing clear conclusions on the effect of a post-prandial nap or bright light exposure on task switching under habitual sleep conditions.

In the present study, we therefore compared the impact of a relatively short afternoon nap and of bright light exposure on switch costs during the post-lunch dip in participants following their habitual sleep routine. To maximize potential applications to real-life situations, the total duration of the nap was restrained to 30 minutes, a feasible duration for post-lunch napping during working hours. Bright light was administered using a glasses-like device that can be easily implemented while working, reading and moving around.

## Material and Methods

### Participants and Protocol

The protocol was approved by the Ethic Committee of the Université Libre de Bruxelles, Belgium. Each participant signed an informed consent form.

Healthy volunteers aged 18-30 years were recruited via advertisements and word of mouth. Potential volunteers contacted the investigators to receive short questionnaires on demographics, sleep habits, depression and chronotype. Exclusion criteria included irregular sleep schedules, bedtimes less than 7 hours or more than 9 hours, smoking on a regular basis, being left-handed, scoring higher than 8 on a modified version of the Beck depression inventory [57] and scoring as extreme morning or evening chronotype on the Morningness-Eveningness questionnaire [58] (see the flow diagram of recruitment and screening in S1 Fig).

Nap and bright light interventions were examined using a parallel group design, and each intervention included its own control condition in a within-subject design. The two groups were run independently from one another. For 3 days prior to each condition, participants were required to comply with regular bedtimes, according to their habitual sleep-wake schedules. Wrist activity (Actiwatch, MiniMitter Inc.) and sleep logs were monitored to ensure compliance.

**Fig 1:**
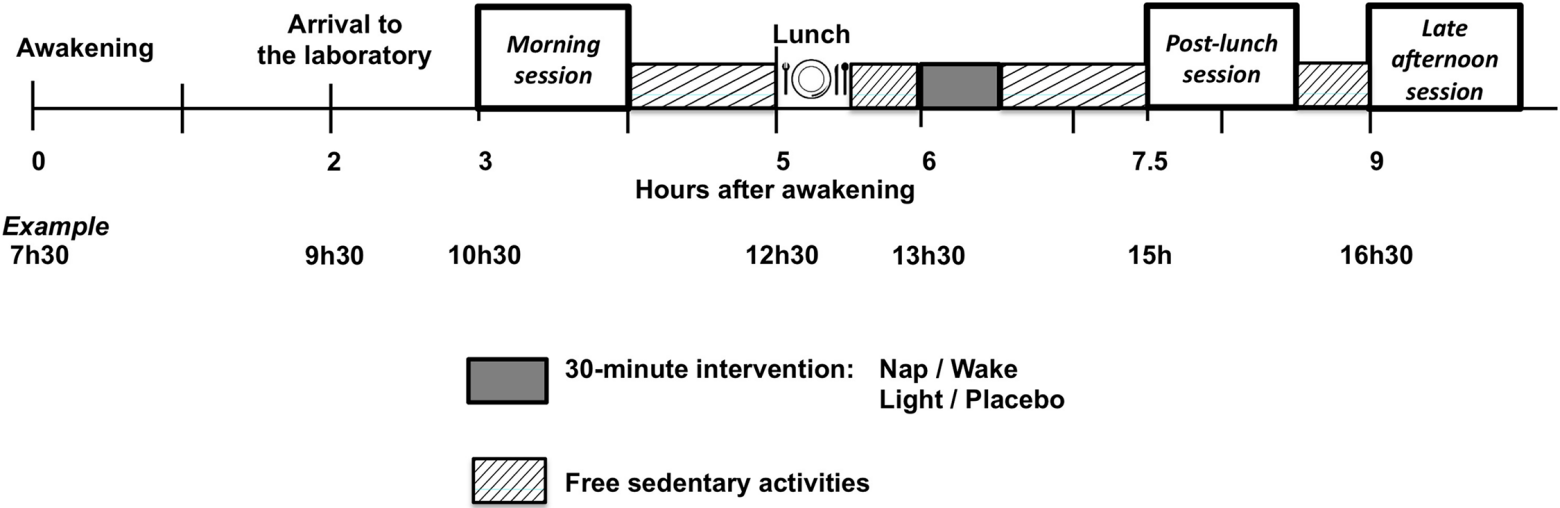
Schematic representation of one session (wake or nap, bright light exposure or placebo) of the protocol. Each pair of sessions was scheduled according to the participant’s sleep schedule and wake up time. An example of the timings is shown for a person waking up at 7:30am.

Each participant was scheduled for 2 randomized conditions (either nap and wake, or bright light and placebo) separated by at least one week. Fig 1 represents the time frame for one of the conditions. The exact schedule was defined based on each participant’s wake up time (Fig 1 shows an example for a person waking up at 7h30). After waking up on the third day of wrist activity monitoring, participants arrived 2 hours later to the research laboratory where he/she spent the entire day. Three testing sessions were programmed 3 (morning or baseline), 7.5 (post-lunch session, one hour after the intervention ended) and 9 (late afternoon session, 2.5 hours after the intervention ended) hours after waking up in the morning. Five hours after the morning waking-up, a lunch was served and, one hour later, the 30-minute intervention occurred. The lunch consisted of an identical portion of lasagna in all study conditions. To keep as close as possible to real world conditions, participants stayed in a room with a light intensity at eye level < 300 lux, except for the intervention period. They kept themselves busy by talking to the study coordinator, studying, reading and working on the computer when they were not tested. They were required to stay seated except for bathroom breaks. The four interventions occurred in an adjacent room.

Electro-encephalographic, -oculographic, and -myographic recordings were obtained during each intervention. Electrodes were located at central (C3-C4), occipital (O1-O2) and frontal (Fz) leads on the scalp, on the right and left outer canthi (eye movements) and the chin. In the nap condition, sleep stages were determined using standard criteria [59]. In the nap/wake condition, each participant either stayed awake for 30 minutes, in a sitting position, while watching a documentary or was required to nap for 30 minutes, lying in bed, in a quiet, dark room. In the bright light/placebo condition, each participant watched a documentary for 30 minutes, in a sitting position, while wearing either a Luminette (Lucimed SA, Belgium) or a placebo Luminette (Lucimed SA, Belgium). Both devices are portable and have the exact same appearance. Light is directed to the lower part of the retina. The difference between the Luminette and the placebo Luminette is the light intensity and wavelength, i.e. a white light enriched with blue light in the range of 460 nm for the Luminette and a white light enriched with orange light in the range of 600 nm for the placebo Luminette. During the 30-minute intervention, the light intensity at eye level was < 70 lux during the wake condition, < 200 lux under the dim orange light condition and about 2000 lux under the blue bright light condition on a background of <70 lux for both light exposure conditions. The nap intervention occurred in complete darkness.

### Cognitive measures

Each testing session consisted of the administration of questionnaires and tasks. Bi-directional visual analog scales for both fatigue (VAS-F) and sleepiness (VAS-S) were completed (one labelled from ‘very little’ to ‘a lot’ and vice-versa). The scores of the two bi-directional scales for fatigue were then averaged for each participant. The same procedure was used for the VAS-S. Vigilance was assessed using a 10-minute Psychomotor Vigilance Task (PVT [60]). In the PVT, participants were instructed to press a key as fast as possible whenever a millisecond countdown appeared in the middle of the computer screen. Stimuli were randomly presented with an inter-stimuli interval ranging from 2 to 10 seconds.

**Fig 2:**
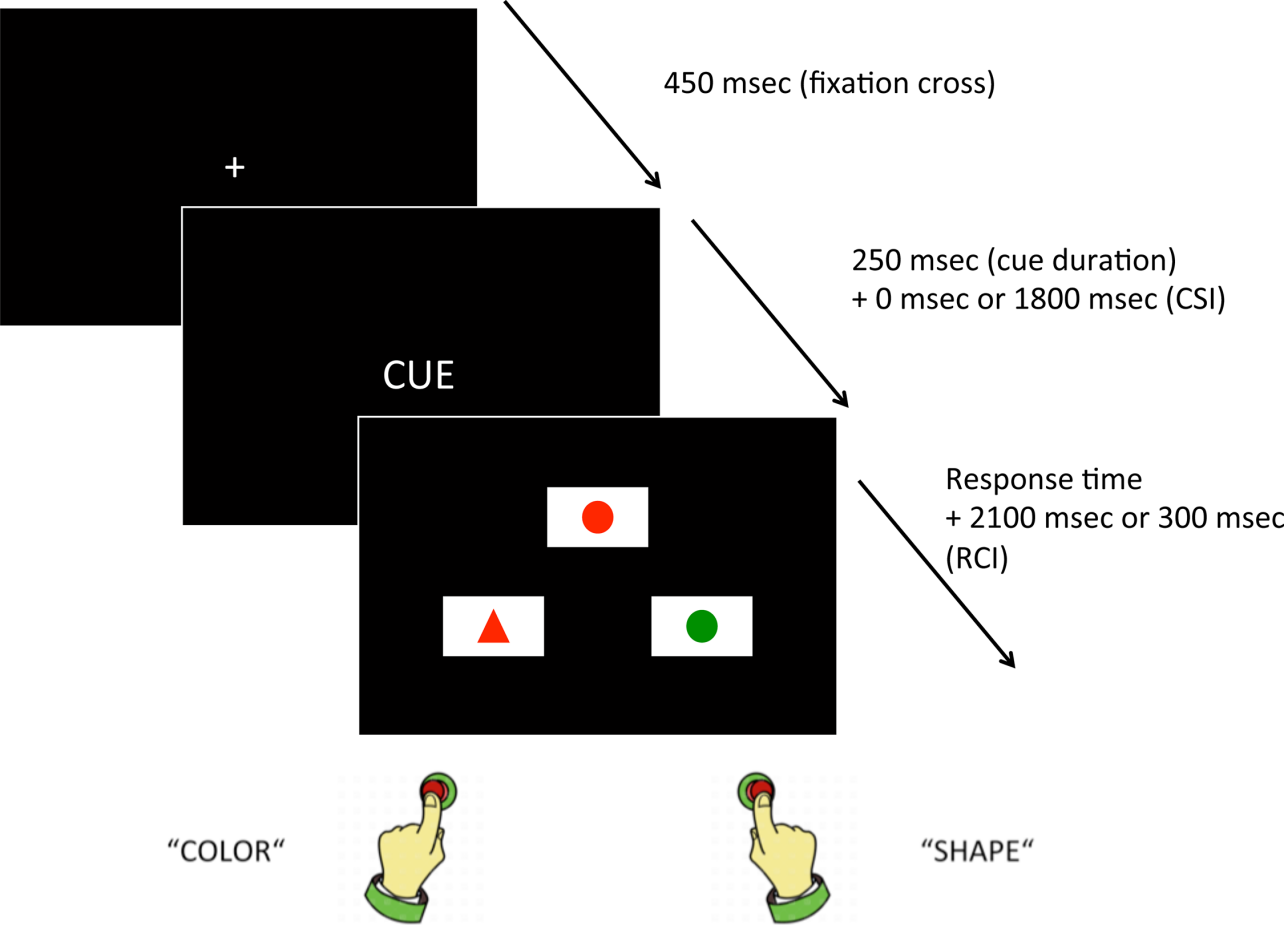
Switching task. For each trial, a fixation cross is followed by a cue, after which three figures are presented. Participants are asked to decide which of the 2 lower figures matches the top one, depending on the cue, and is required to press the corresponding key (left key for left figure and right key for right figure).

Task switching was then assessed using a cued match-to-sample task developed in our laboratory (Fig 2). Investigating proactive and reactive processes in task switching requires an adapted procedure. Among task-switching procedures, the cuing procedure [48] presents a series of targets upon whose presentation one of the tasks has to be performed. On each trial, a task cue indicates which task must be executed. The switch cost is measured by comparing transitions involving a task switch (switch trials) with transitions involving a task repetition (repeat trials) in a single sequence of events. A better control of the time intervals is a major advantage of the cuing procedure. Due to the occurrence of a cue, the response-stimulus interval (RSI) is subdivided into a response–cue interval (RCI) and a cue–stimulus interval (CSI), that is, RSI = RCI + CSI. Consequently, RCI and CSI can be manipulated independently. With a longer RCI, the activation of the previous task set may decay (also known as task-set dissipation). With a longer CSI, time available for preparation for the next task before stimulus onset increases [45]. A long CSI therefore allows proactive control to take place while a short CSI mainly involve reactive control. To control for task duration, the RSI is kept constant between short and long CSI conditions. The RCI is thus longer with short CSI and shorter with long CSI. In the cued match-to-sample task, at the beginning of each trial a cue appeared in the center of the screen. Participants were then required to match a stimulus (presented in the upper part of the screen) to one of two stimuli (presented in the lower part of the screen). Responses were always spatially congruent to the target location, i.e. left key for left answer and right key for right answer. Short and long CSI were presented to evaluate the reactive and proactive components of task switching [46,47].

The switching task was programmed using Psyscope X software [61–63] and run on Imac G5 computers. Participants answered on the computer's keyboard using right and left hand forefingers. Each reaction time (RT) was recorded as the response time from target onset to the nearest millisecond using the computer internal clock. The cue was the written word of the relevant dimension (in French): *COULEUR* (color), *FORME* (shape), *NOMBRE* (number) and *CONTOUR* (outline). The cued match-to-sample task allows controlling for congruency effects. Each trial involves two dimensions that differ (e.g. shape and color) and two dimensions that are kept constant (e.g. number and outline). Trials are all incongruent ones (i.e. each dimension is associated with a different response) so that it is always possible to know if the participant responds correctly or not according to the target dimension. Incongruency is, thus, kept constant during the entire task. Therefore, as conflicting dimensions are present within the same object (as in a classical Stroop task), an inhibitory component is always present in both repeat and switch trials. Stimuli were presented on a black screen within three rectangles [9 cm wide (visual angle = 8.58°) and 5 cm tall (4.77°)] in a triangular disposition, with the target picture on the top. The geometrical figures were 2.5 cm wide (2.39°) and 1.5 cm tall (1.43°). The distance between the screen and the participants' eyes was approximately 60 cm.

Prior to the task, participants read the instructions specifying to press a key ("q" for left and "m" for right on an azerty keyboard) corresponding to the position (left or right, respectively) of the bottom picture that matched the target (top picture) according to the cued dimension. A sample trial figure was included as an example. The participants were instructed to perform as fast as possible while minimizing the number of errors. Each trial began with a fixation cross displayed at the center of the screen during 450 ms, followed by the cue presented for 250 ms. Short and long CSI conditions were presented in a counterbalanced order across participants. Beside the warm-up trials, in each CSI, there were two blocks of 96 trials (48 switch and 48 repeat trials in each block). In the short CSI condition, the cue was directly followed by the target and the two potential matching figures until a response was provided. In the long CSI condition, a black screen followed the cue during 500 ms and was replaced by a point in the center of the screen for 250 ms. This sequence (black screen + point) was presented twice, followed by another black screen for 300 ms, before the target and the two potential matching figures appeared. The CSI was thus 0 ms in the short CSI condition and 1800 ms for the long CSI condition. The RCI was 300 ms in the long CSI condition and 2100 ms in the short CSI condition to balance the total time for each trial. Both short and long CSI were preceded by a training block of 10 trials and were administered in a single session that lasted approximately 30 minutes, with a short break between each interval type.

### Data analysis

Data are expressed as mean ± SD unless otherwise indicated. The scores obtained on the two visual analog scales for both sleepiness and fatigue were averaged before statistical calculations. From the PVT data, tonic alertness was evaluated using the average of the RTs between 100 and 500 ms, 10% fastest RTs, 10% slowest RTs and RT variability that was computed as the difference between the 10% slowest and the 10% fastest RTs [5].

One participant in the bright light/placebo group was eliminated because no data on task switching was obtained due to technical failure. Task switching analyses are based on RTs for trials with correct responses only (task-switching speed), and on percentages of correct responses (task-switching accuracy). The first three trials in each block were considered as warm-up trials and excluded from the analyses. Trials following an error were also discarded (6% of trials). RTs' outliers were identified for each subject, CSI duration (short vs. long) and task switching conditions (repeat vs. switch trials) using the generalized extreme studentized deviate (GESD) test [64] with an *r* limited to 20% of the data. This procedure led to discard 2.3% of trials. Latency switch-cost score was computed by subtracting the mean RTs during repeat trials from the mean RTs during switch trials. Accuracy switch-cost score was calculated by subtracting the percent of correct responses during switch trials from the percent of correct responses during repeat trials. Consequently, an increase in switch-cost is always represented by a positive value and indicates a worsened performance.

For comprehensive analyses of switch costs within the three sessions, see Supporting Information (S1 Text). Changes in questionnaire scores and task (PVT and task switching) performances from baseline were estimated by subtracting the variables obtained during each of the two afternoon sessions from those obtained during the morning session [post-lunch session - baseline (ΔPL); late afternoon session - baseline (ΔLA)]. These differences were submitted to repeated measures ANOVAs with Session (ΔPL vs. ΔLA) and Intervention (control condition vs. intervention) as within-subject factors, and Group (bright light vs. nap) as between-subject factor. The within-subject factor Preparation Time (short vs. long CSI) was also included when performance in task switching was tested. Interactions were analyzed using Tukey’s post-hoc comparisons. In addition, one sample *t*-tests against 0 for ΔPL and ΔLA were used to assess significant differences between the morning session and the subsequent ones (Cohen's d is indicated and reflects the effect size). Correlation analyses were performed on differences between interventions and control conditions for each variable of interest.

Significance level was set at *p*<0.05 (two-tailed). A *p*-value between 0.05 and 0.10 refers to a trend. *P*-values in ANOVAs were adjusted in the post-hoc tests using Tukey’s correction for multiple comparisons. *P*-values were adjusted using Bonferroni correction per variable that was compared to the measures of task switching: correlations between task switching and PVT were corrected for the four PVT measures (average of RTs, 10% highest RTs, 10% lowest RTs and RT variability), correlations between task switching and sleep stages were corrected for the 2 sleep stages taken into account (N1 and N2 stages).

## Results

### Participants

Twenty-five young healthy volunteers participated in the study: 15 participants in the nap/wake group (12 women, mean ± SD: 22.1 ± 2.2 years old, body mass index: 22.8 ± 4.1 kg/m^2^), 10 participants in the light/placebo group (8 women, 23.4 ± 1.6 years old, 20.5 ± 1.6 kg/m^2^). At screening, depression scores averaged 1.5 ± 2.1 in the nap/wake group and 1.8 ± 1.5 in the light/placebo group, indicating low depression scores, below cut-off levels. Circadian typology averaged 51.1 ± 8.2 in the nap/wake group and 49.5 ± 4.6 in the light/placebo group, indicating neutral to moderate evening or morning chronotypes.

### Sleep variables

In the nap intervention group, sleep duration evaluated by wrist activity recordings over the 3 nights were similar in both conditions (wake condition: 7h06min ± 54min and nap condition: 7h10min ± 47min, *P* = 0.77), as well as in the light intervention group (placebo condition: 6h52min ± 49 min and light condition: 6h53min ± 50 min, *P* = 0.99).

Total sleep time during the 30-minute nap opportunity averaged 13.2±10.1 minutes, with 6.3±5 minutes of stage N1 and 6.7±9 minutes of stage N2. No REM was detected during the nap. One participant obtained 0.5 minute of stage N3, and two did not sleep at all. No sleep stage was detected in the three remaining conditions.

Further analyses were performed without the 2 participants who were not able to achieve any sleep during the nap condition.

### Sleepiness, fatigue and alertness

Repeated measures ANOVA on VAS-S scores with factors Group (bright light vs. nap), Intervention (intervention vs. control condition) and Session (ΔPL vs. ΔLA) revealed a significant Group x Session interaction [*F*(1,20) = 9.75, *P* = 0.005]. Post-hoc comparisons indicated that, in the nap group, sleepiness increased more in late afternoon compared to post-lunch session (+1.2 ± 1.2 cm vs. +0.3 ± 1.1 cm, *P* = 0.01). One-sample *t*-tests against the constant value 0 showed a significant increase of sleepiness in late afternoon [*t*(12) = 3.1; *P* = 0.009; d *=* 1] but not during the post-lunch session [*t*(12) = 1.04; *P* = 0.317]. Analyses conducted on VAS-F and PVT variables did not reach statistical significance (all *P*s > 0.11) except for a significant effect of Session x Group for 10% fastest RTs [*F*(1,20) = 5.8; *P* = 0.026]. Post-hoc comparisons indicated a significant difference in ΔLA with participants in the nap group being slower in late afternoon compared to baseline, whereas participants in the bright light group were faster (+7.74 ± 13.35 vs. -7.28 ± 17.16 msec, *P* = 0.029). Neither main effect of Intervention nor interaction with this factor reached statistical significance.

### Task-switching speed (latency switch-cost score)

**Fig 3:**
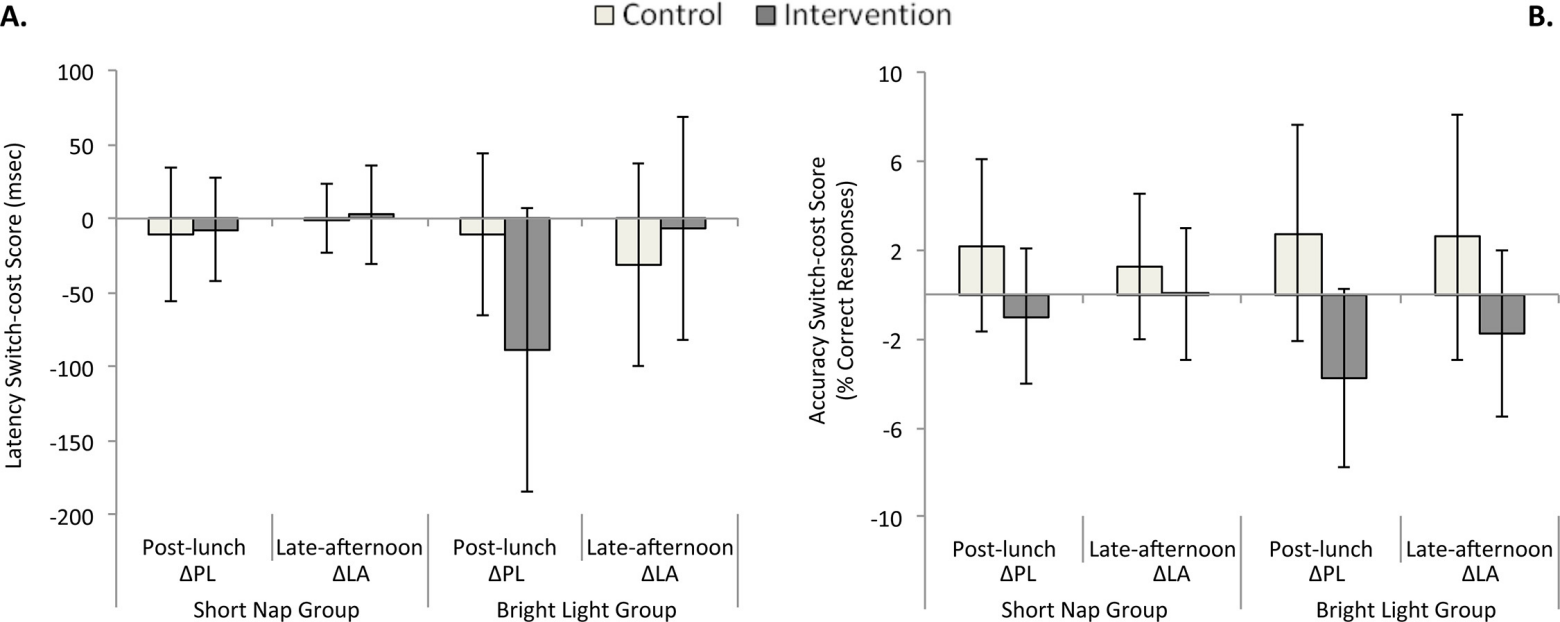
Switch costs. Latency switch-cost score (reaction times, Fig 3A) and accuracy switch-cost score (correct responses, Fig 3B) are represented for both groups (nap/wake and bright light/placebo). ΔPL (ΔLA) indicates the difference of switch cost between the morning session and the post-lunch session (late afternoon session). Vertical bars represent SD. Bright light intervention resulted in a decreased latency switch-cost score post lunch (Fig 3A). In the control condition (wake/placebo), a post-lunch dip in task switching was evidenced by an increased accuracy switch-cost score. Both interventions (short nap/bright light) elicited a significant decrease of this post-lunch dip (Fig 3B).

Changes of latency switch-cost score are represented in Fig 3A for both groups. Repeated measures ANOVA on changes in latency switch-cost score indicated a significant main effect of Group [*F*(1,20) = 5.3, *P* = 0.032], a significant Intervention x Session interaction [*F*(1,20) = 5.8, *P* = 0.026] and a significant interaction Intervention x Group x Session [*F*(1,20) = 5.86; *P* = 0.025]. Post-hoc comparisons on this latter interaction disclosed a trend for a higher decrease of the switch cost during the post-lunch session after bright light intervention compared to its control condition (bright light: -89 ± 95 ms vs. placebo: -11 ± 54 ms, *P* = 0.051). One-sample *t*-tests against the constant value 0 revealed that the decrease of switch cost post lunch was significant only with bright light intervention [*t*(8) = -2.79; *P* = 0.024; d = 0.94]. Changes in latency switch-cost score also differed between post lunch and late afternoon: bright light intervention was associated with a more pronounced switch-cost decrease post lunch, i.e. a better performance (ΔPL: -89 ± 95 ms vs. ΔLA: -7 ± 75 ms, *P* = 0.039). Altogether, these results suggest a short-term benefit of bright light on latency switch-cost score. The difference in task switching performance between the bright light intervention and its control condition was not correlated with post lunch differences in sleepiness/fatigue/objective alertness (all *P*s > 0.26). Neither the main effect of Preparation Time nor any interaction with this factor reached statistical significance (all *P*s > 0.10).

### Task-switching accuracy (accuracy switch-cost score)

Changes of accuracy switch-cost score are represented in Fig 3B for both groups. Nine out of 14 subjects in the nap group and 6 out of 9 subjects in the bright light group showed a post-lunch dip in accuracy switch-cost score.

Repeated measures ANOVA on changes in accuracy switch-cost score revealed a main effect of Intervention [*F*(1,20) = 12.12, *P* = 0.002] indicating that both interventions elicited a decrease in accuracy switch-cost score. One-sample *t*-tests against the constant value 0 showed a decrease of switch cost after both interventions [-1.42 ± 3.13%, *t*(21) = -2.13; *P* = 0.045; d = 0.45] whereas task-switching accuracy worsened under control conditions (wake/placebo) with an increase of switch cost [+2.11 ± 3.89%, *t*(21) = 2.54, *P* = 0.019, d = 0.54].

**Fig 4:**
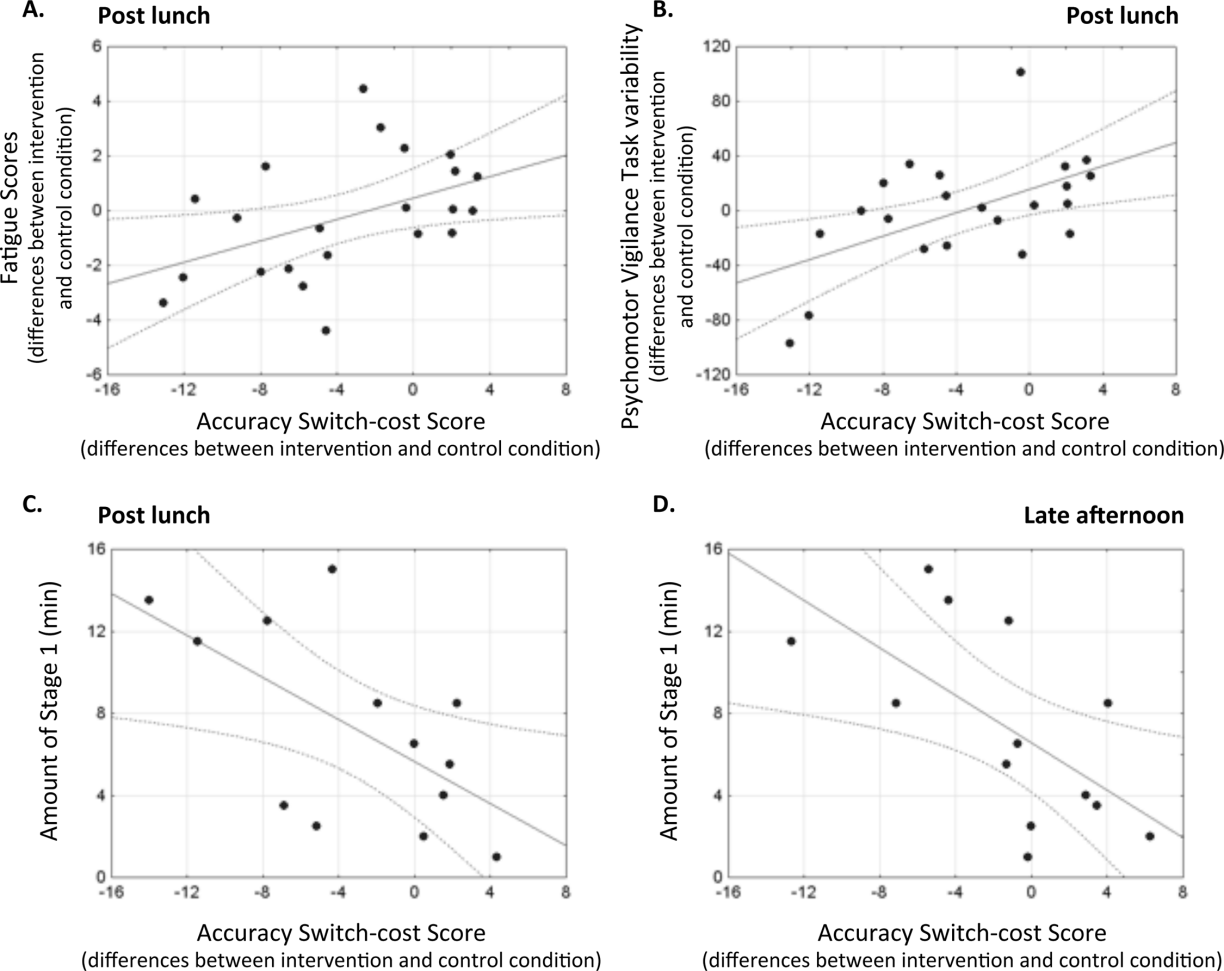
Correlations. Significant correlations were obtained for the modulations (differences between intervention and control conditions) between A. the accuracy switch-cost score and the fatigue scores during the post-lunch session (Fig **4**A, both groups), B. the accuracy switch-cost score and the variability in the Psychomotor Vigilance Task during the post-lunch session (Fig **4**B, both groups), C. the accuracy switch-cost score post lunch and the amount of stage N1 (Fig **4**C, nap group only) and D. the accuracy switch-cost score in the late afternoon and the amount of stage N1 (Fig **4**D, nap group only).

When differences between intervention and control conditions were examined, a significant correlation was observed between the modulation of accuracy switch-cost score and the modulation of VAS-F scores during the post-lunch session (*r* = 0.46; *P* = 0.03, Fig 4A), indicating that the participants who felt less tired after the intervention also exhibited a lower switch cost. In addition, the modulation of the PVT variability during the post-lunch session was also correlated with the accuracy switch-cost score modulation (Fig 4B, *r* = 0.55; *P* = 0.008), indicating that participants with a higher decrease of switch cost were those with less variability in their PVT performance post lunch. All other correlations between switch-cost modulations and differences in sleepiness/fatigue/objective alertness were not significant (coefficients of correlation range from -0.26 to 0.42; all *P*s > 0.051). Correlations between the duration of sleep stages N1 and N2 during the nap and switch-cost modulations revealed a negative association between the amount of stage N1 and differences between the nap intervention and its control condition in accuracy switch-cost score post lunch (Fig 4C, *r* = -0.62, *P* = 0.025) and in the late afternoon (Fig 4D, *r* = -0.63, P = 0.020). This indicates a higher decrease in switch cost post lunch and in the late afternoon for the participants who achieved more minutes of stage N1 during the nap. Correlations between the duration of stage N2 during the nap and switch-cost modulations were not significant (coefficients of correlation range from 0.12 to 0.22; all *P*s > 0.47).

The interaction Intervention x Session in the ANOVA on switch cost accuracy was marginally significant [*F*(1,20) = 4.1, *P* = 0.056]. Post-hoc comparisons showed a difference between interventions and control conditions post lunch (-2.13 ± 3.64% vs. 2.41 ± 4.2%, *P* < 0.001) and in the late afternoon (-0.71± 3.35% vs. 1.8 ± 4.28%, *P* = 0.009). One sample *t*-tests against the constant value 0 for ΔPL indicated a significant switch-cost increase post lunch in control conditions for both groups [+2.41 ± 4.2, *t*(21) = 2.691; *P* = 0.014; d = 0.57] and a significant switch-cost decrease after both interventions [-2.13 ± 3.64, *t*(21) = -2.739; *P* = 0.019; d = 0.59]. However, increased switch-cost in control conditions (tested with one-sample *t*-tests against constant value 0 for ΔLA) was only marginally significant for the late afternoon sessions [+1.81 ± 4.28, *t*(21) = 1.981; *P* = 0.061; d = 0.42] and decreased switch-cost failed to reach statistical significance after both interventions [-0.71 ± 3.36, *t*(21) = -0.996; *P* = 0.331; d = 0.21]. These results demonstrate a post-lunch dip in task-switching accuracy that is reversed by both countermeasures (bright light exposure and napping).

Neither the main effect of Preparation Time nor any interaction with this factor reached statistical significance (all *P*s > 0.10).

## Discussion

The current study investigated the impact of a 30-minute afternoon nap opportunity or bright light exposure on task switching, both post lunch and in late afternoon. Although cognitive flexibility was decreased post lunch in control conditions, subjective sleepiness and PVT performance remained unaffected. One peculiarity of the present study was that background light level was kept close to real world conditions during the entire protocol (except for the intervention period and during the nap), which rendered the experiment more ecological and stimulating. This might partially explain the post-lunch maintenance of performance in simple vigilance tasks (i.e., the PVT), whereas a post-lunch dip in performance is observed with more demanding higher-order cognitive functions. We actually evidenced a post-lunch dip in task-switching accuracy, which was alleviated by the interventions of both napping and bright light exposure. Moreover, the modulation of task-switching accuracy was associated with both scores of fatigue and variability on a vigilance task during the post-lunch session, indicating that less fatigued and more alert participants reached a higher mental flexibility post lunch. In the nap group, the more the participants spent the nap in stage N1 of sleep, the less they made mistakes during the post-lunch session. In addition, only bright light was efficient to reduce the latency switch-cost score post lunch.

To maximize the feasibility of napping or bright light exposure in real life, we restrained the total duration of the nap to 30 minutes, a duration that can be applied during working hours. To administer bright light, we used a luminotherapy device that can easily be implemented during working hours, as compared to other techniques for which the participants have to stay in the front of the light source. Our study showed more potential benefits of bright light exposure on mental flexibility than a nap, which is a more acceptable countermeasure to set up in a workplace than a nap as it does not require any work interruption.

We did not replicate previous findings that evidenced a larger impact of napping on task switching than bright light [42]. On the contrary, in our study, bright light had a large effect on switch cost, affecting both accuracy and speed. Methodological differences between our experiment and the Kaida et al. [42] study may explain this discrepancy. First, our participants followed their habitual time in bed before both laboratory sessions whereas participants in Kaida study were sleep restricted by 20%, which resulted in a higher sleep pressure on the following day and probably worse performances before any intervention. Second, the participants in our study were in bed for 30 minutes and achieved on average 13.2±10.1 minutes of sleep. In the Kaida et al. study [42], participants slept for 20 minutes but time in bed to achieve these 20-minute asleep is unknown. As several studies indicated that a nap but also a period of rest in a quiet dark room promote cognitive functions compared to busy wakefulness (e.g. auditory learning [65]; implicit memory [66]), different times in bed between participants may be a confounding factor. Our choice of restricting the time in bed for the nap to 30 minutes is justified by a reasonable duration for napping during working hours. Third, the discrepancy in the results of the two studies might be due to an improved efficacy of the bright light administration in our study that used a portable device vs. ambient bright light in the Kaida et al. study [42]. Further studies are needed to compare the beneficial effects of ambient bright light vs. luminotherapy technology on task switching and on executive functions in general. Fourth, it should also be noticed that lunchtime was scheduled at 11.00am in the Kaida et al. study [42], a quite unusual timing to eat lunch. Lastly, our switching task focused on task-goal activation rather than task rules. Benefits of our interventions therefore occurred at the task-goal level and did not involve long-term learning such as complex S-R associations or rehearsal of such associations [67].

The duration of sleep needed to initiate the cascade of neurophysiological events that would eventually lead to improved sleepiness, fatigue and executive functioning remains an open question. In the current study, the average amount of sleep achieved by our participants during the nap was relatively low (about 13 minutes) with 6 minutes of stage N1 and 7 minutes of stage N2. Tietzel and Lack [68] have shown an improvement of alertness and cognitive performance (Symbol–Digit Substitution Task and Letter Cancellation Task) after a 10-minute nap, as compared to a similar period of time awake, but not after a 30sec or a 90sec nap, indicating that the benefits of a nap are not initiated by the onset of stage N1 sleep. Despite a different choice of tasks, our study is in line with this concept as we found a negative association between the amount of stage N1 and accuracy switch-cost score, i.e. the more the participants spent their nap in stage N1, the more they reduced their accuracy switch-cost score. Of note, when the 2 participants who did not sleep during the nap period were included in these correlations, the findings remained similar (see S2 Fig). To the best of our knowledge, our study is the first to evidence an association between stage N1 duration and a lower post-lunch dip in mental flexibility.

A decrease in switch cost was present for both short and long intervals (CSI), suggesting an improvement in reactive control processes, rather than a modulation of proactive control [46,69]. Indeed, a specific modulation of switch cost with a long CSI should be expected for proactive control modulation. Our results did not evidence any specific modulation of switch cost according to CSI, which could suggest a slight, if any, modulation of proactive control by our interventions but rather an influence of reactive control. The association of decreased switch cost with improved vigilance and lower subjective fatigue is also compatible with a reactive control, rather than a proactive one. Indeed, reactive control is characterized by transient activation of lateral PFC, along with a wider network of additional brain regions potentially involved in vigilance and fatigue. This transient activity might reflect task goals reactivation through bottom-up processes [46].

Beside small sample sizes that differ between the two groups, another potential limitation of our study is the lack of control for the amount of cognitive challenge between testing sessions. However, the time allocated for free activities was very limited. Indeed, the participants were allowed to engage in sedentary activities such as reading, watching movies, working on the computer four times per condition (between the morning session and the lunch, between the lunch and the intervention, between the intervention and the post-lunch session and between the post-lunch session and the late afternoon session), from 30 to 60 minutes.

To conclude, we found a post-lunch dip and a late afternoon decrease in mental flexibility in well-rested participants. Exposure to bright light resulted in similar beneficial effects than a short nap on task-switching accuracy, both post lunch and in late afternoon. In addition, bright light exposure was associated with a reduction of the switch cost in response speed post lunch. Future research is needed to investigate the detrimental consequences of total or partial sleep restriction on cognitive flexibility during the post-lunch dip using the same task-switching paradigm.

## Supporting Information

S1 Dataset Raw data. (XLSX)

S1 Fig Flow diagram of recruitment and screening. (TIF)

S2 Fig Correlations with all participants. Correlations between A. the accuracy switch-cost score post lunch and the amount of stage N1 (nap group only, with all participants) and B. the accuracy switch-cost score in the late afternoon and the amount of stage N1 (nap group only, with all participants).
(TIF)

S1 Text Supplementary analyses. Comprehensive analyses of switch costs within the three sessions.
(PDF)
