# Supplementary figures and images for: Afternoon Nap and Bright Light Exposure Improve Cognitive Flexibility Post Lunch
Source: PLoS One. 2015 May 27;10(5):e0125359. doi: 10.1371/journal.pone.0125359 (PMC4446306; doi:10.1371/journal.pone.0125359)

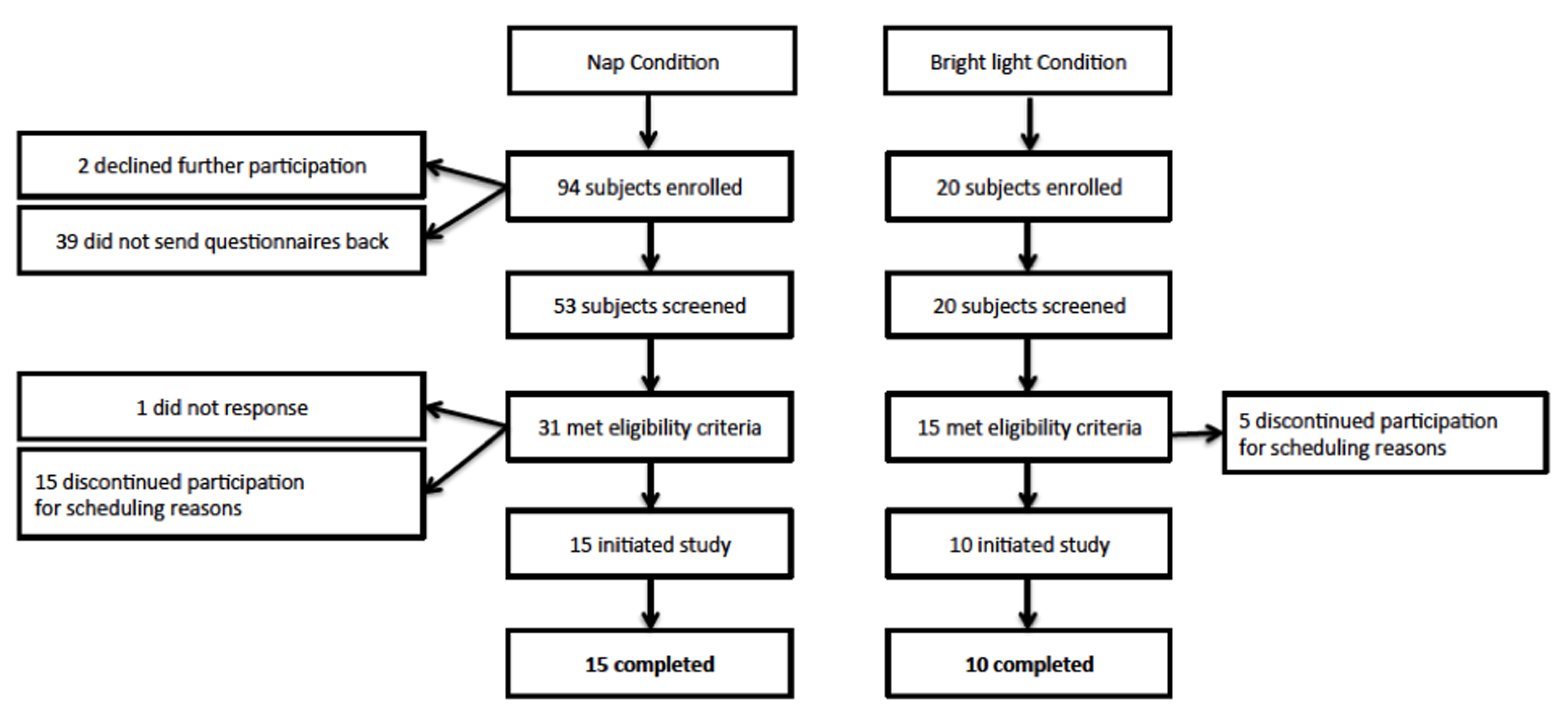

Supplement: S1 Fig — (TIF) [file pone.0125359.s002.tif]

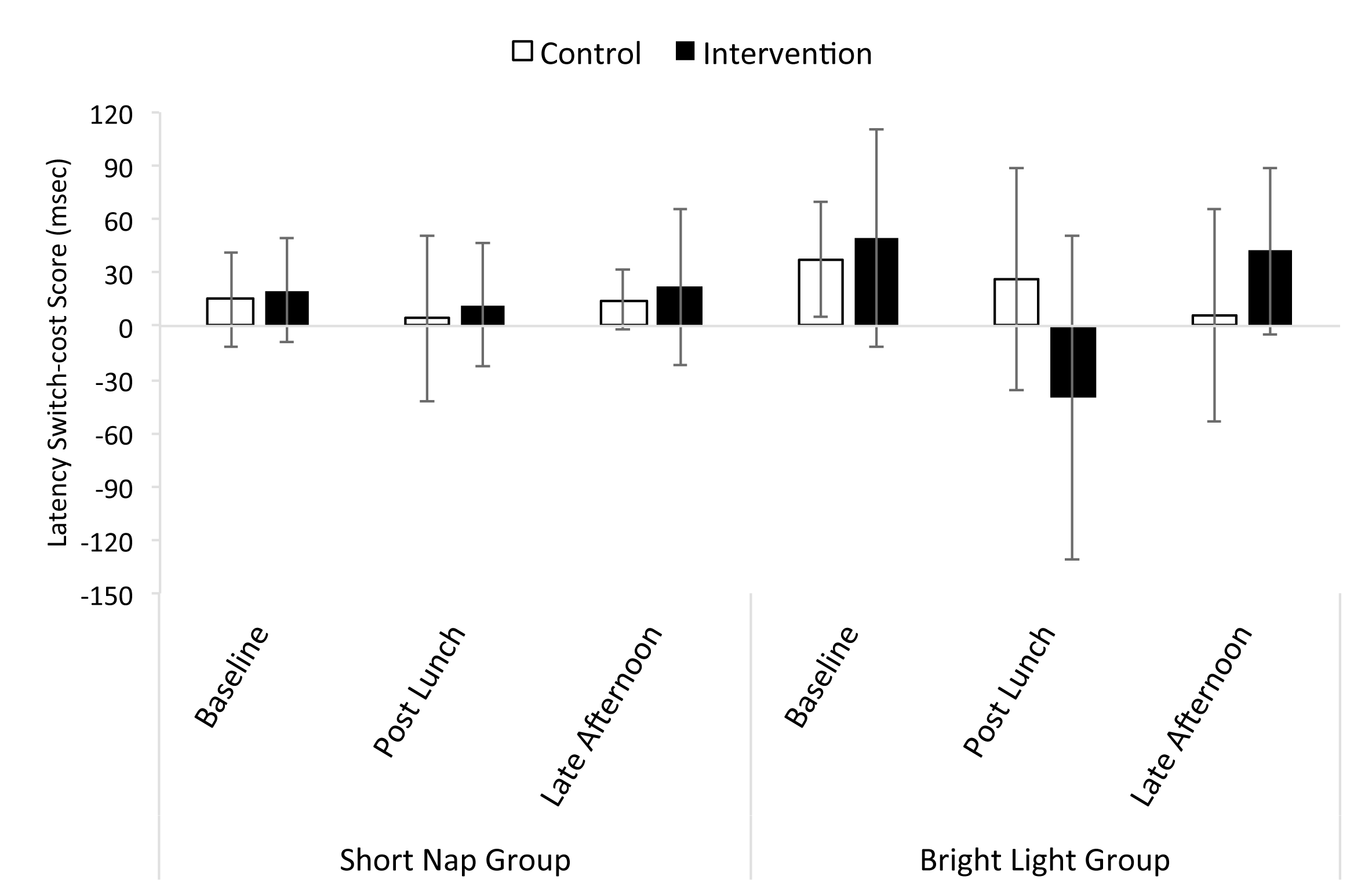

Supplement: S2 Fig — Correlations between A. the accuracy switch-cost score post lunch and the amount of stage N1 (nap group only, with all participants) and B. the accuracy switch-cost score in the late afternoon and the amount of stage N1 (nap group only, with all participants). (TIF) [file pone.0125359.s003.tif]
