# Supplementary material for: Afternoon Nap and Bright Light Exposure Improve Cognitive Flexibility Post Lunch
Source: PLoS One. 2015 May 27;10(5):e0125359. doi: 10.1371/journal.pone.0125359 (PMC4446306; doi:10.1371/journal.pone.0125359)
Supplement: S1 Text — Comprehensive analyses of switch costs within the three sessions. (PDF) [file pone.0125359.s004.pdf]

Supporting Information

S1 File. Supplementary analyses

Data analysis

Both switch costs and raw data (i.e. with repeat and switch trials) were submitted to repeated measures ANOVAs with Session (morning vs. post-lunch vs. late afternoon) and Intervention (control condition vs. intervention) as within-subject factors, and Group (bright light vs. nap) as between-subject factor. The within-subject factor Preparation Time (short vs. long CSI) was also included. For raw data, the within-subject factor Task Switching (repeat trials vs. switch trials) was also included. Interactions were analyzed using planned comparisons to investigate targetted modulations between sessions. The morning session was compared to both post-lunch and late-afternoon sessions. For raw data, interactions were decomposed using Tuckey post-hocs. To assess potential baseline differences, the morning session was compared between groups and intervention conditions. Significance level was set at $p<0.05$ (two-tailed). Correction of the alpha level for multiple comparisons (*P*corr) is presented when needed.

Response latencies

All data are presented in S1 Table. Switch-costs are represented in S3 Fig. for both groups. Raw data are represented in S4 Fig.

Repeated measures ANOVA on latency switch-cost scores indicated a significant Intervention x Session interaction [$F(2,40) = 4.38$, $P = 0.019$] and a significant interaction Intervention x Group x Session [$F(2,40) = 4.57$; $P = 0.016$]. Planned comparisons on this

latter interaction disclosed a significant decrease of the switch cost during the post-lunch session compared to the morning, after bright light intervention compared to its control condition ($P = 0.008$; Pcorr = 0.032). There was no significant modulation of latency switch-cost scores between morning and late-afternoon sessions, after bright light intervention compared to its control condition ($P = 0.283$; Pcorr > 0.999), nor in the nap group between sessions and conditions (all Ps > 0.897; all Pscorr > 0.999). All main effects or other interactions were non significant (all Ps > 0.10).

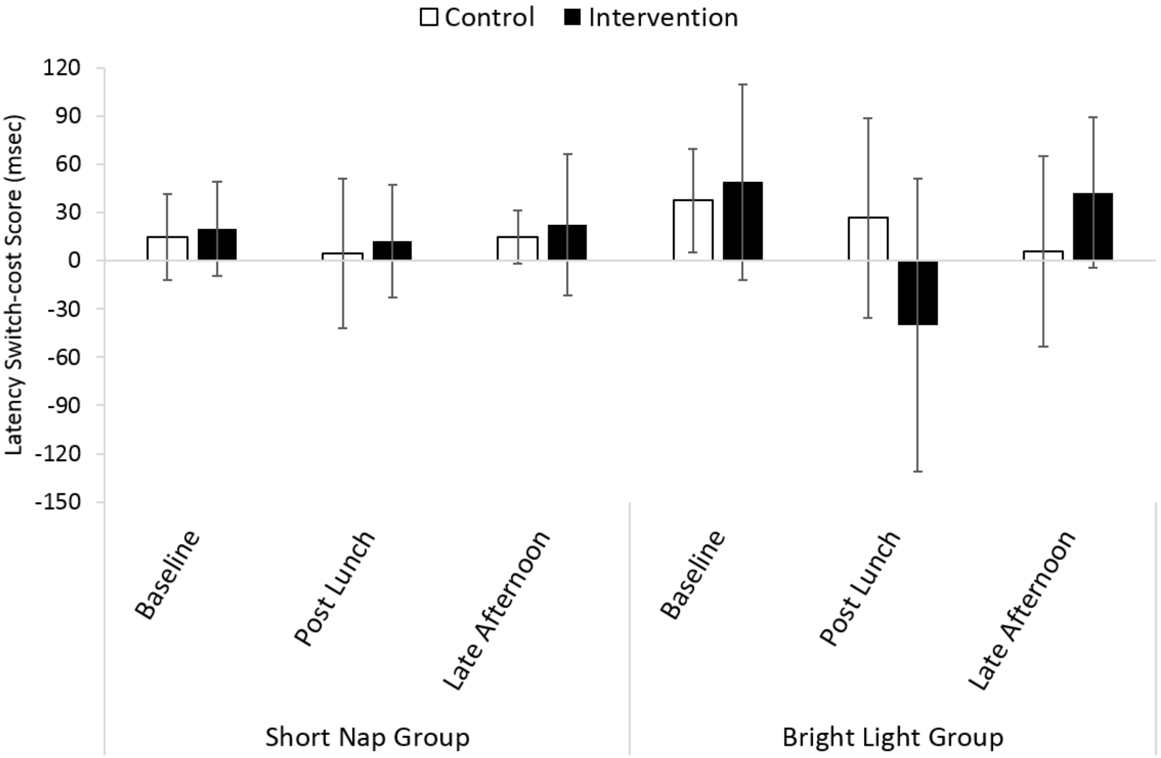

S3 Fig. Latency switch-scores for each group.

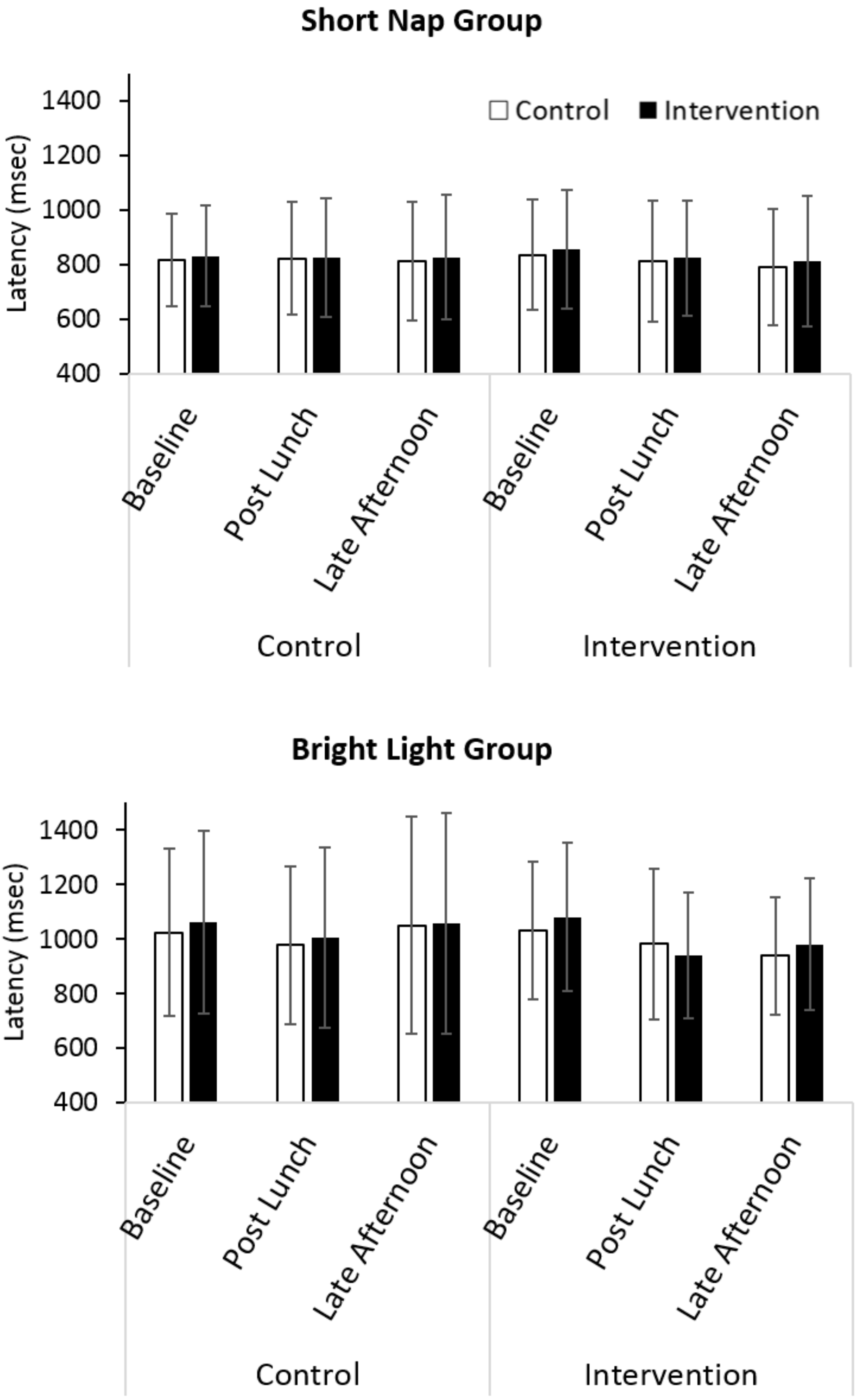

S4 Fig. Repeat and switch trials latencies for each group.

Post-hoc analyses on raw data indicated a significant difference between morning and post-lunch sessions after bright light intervention for switch trials ($P < 0.001$) but not for repeat trials ($P > 0.05$). Participants were faster post-lunch on switch trials. Furthermore, switch-trial latencies were significantly shorter during post-lunch after bright light intervention than after placebo ($P = 0.007$).

The morning session (baseline) did not differ significantly between groups and interventions [$F(1,20) = 0.12$, $P = 0.737$].

S1 Table. Latencies and latency switch-cost scores for each group

	Wake			Nap			Placebo light			Bright light		
	Repeat	Switch	SC	Repeat	Switch	SC	Repeat	Switch	SC	Repeat	Switch	SC
Morning	817 (65)	832 (71)	15 (8)	837 (62)	856 (68)	19 (12)	1024 (78)	1061 (84)	37 (10)	1032 (74)	1081 (81)	49 (15)
Post lunch	822 (67)	826 (74)	4 (15)	812 (67)	824 (62)	12 (18)	979 (81)	1005 (89)	26 (18)	982 (80)	941* (74)	- 41* (21)
Late afternoon	812 (84)	826 (86)	14 (11)	790 (59)	812 (67)	22 (13)	1051 (101)	1056 (104)	5 (13)	938 (71)	980 (80)	42 (15)

Latencies and latency switch-cost scores for each group in msec.

Accuracy

All data are presented in S2 Table. Switch-costs are represented in S5 Fig. for both groups. Raw data are represented in S4 Fig.

Repeated measures ANOVA revealed a significant Intervention x Session interaction on switch cost accuracy [$F(2,40) = 9.02$, $P < 0.001$]. Planned comparisons on this latter interaction disclosed a significant decrease of the switch cost during the post-lunch session compared to the morning, after intervention compared to its control condition (P <0.001; Pcorr = 0.002). A marginally significant decrease of switch cost was also present between

morning and late-afternoon sessions, after intervention compared to its control condition (P = 0.029; Pcorr > 0.058). All main effects or other interaction were not significant (all Ps > 0.10).

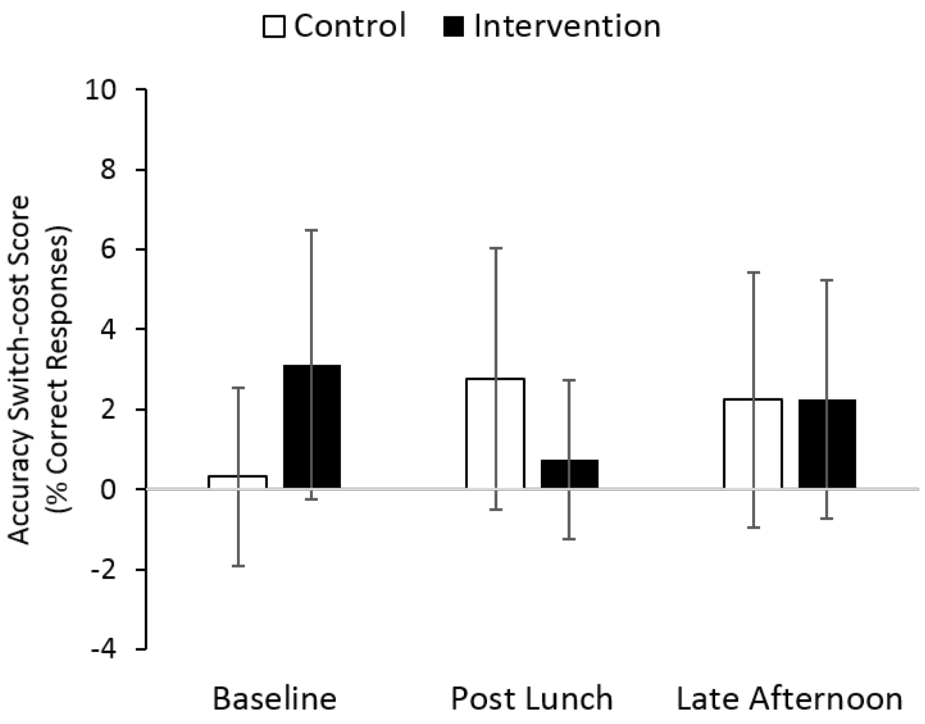

S5 Fig. Accuracy switch cost-scores.

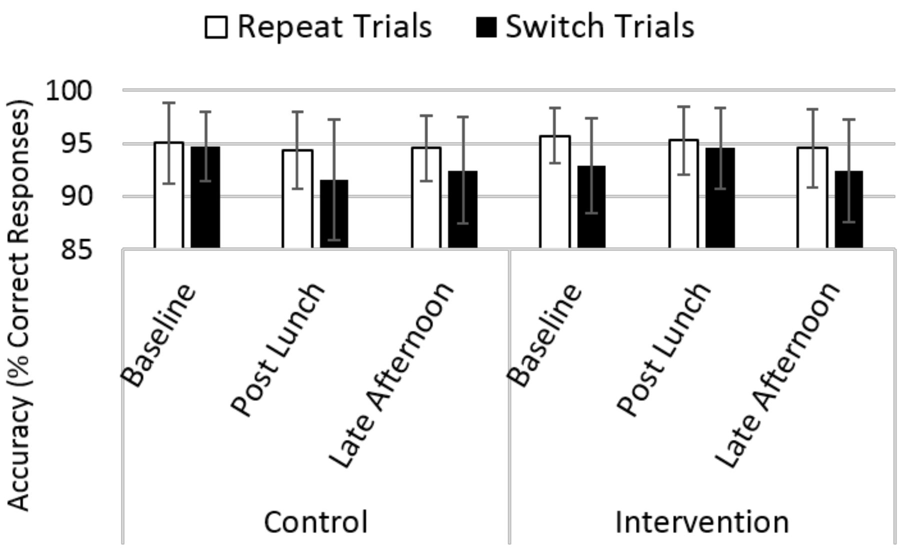

S6 Fig. Repeat and switch trials accuracy.

Post-hoc analyses on raw data indicated that switch-trial accuracy was significantly higher during post-lunch after intervention than after its control condition ($P < 0.001$). Switch-trial accuracy did not differ significantly during late afternoon after intervention compared its control condition ($P = 0.001$). Repeat trials did not differ significantly ($P > 0.10$).

Notably, the morning session (baseline) differed significantly between intervention and its control condition [$F(1,20) = 11.15$, $P = 0.003$]. Switch costs were higher during the morning session in the intervention compared to its control condition.

S2 Table. Accuracy and accuracy switch-cost scores.

	Control			Intervention		
	Repeat	Switch	SC	Repeat	Switch	SC
Morning	95<br>(0.8)	94.7<br>(0.7)	0.3<br>(0.5)	95.9<br>(0.6)	92.7<br>(1)	3.1<br>(0.7)
Post lunch	94.4<br>(0.8)	91.6<br>(1.3)	2.8<br>(0.7)	95.3<br>(0.7)	94.5<br>(0.9)	0.7<br>(0.4)
Late afternoon	94.6<br>(0.7)	92.4<br>(1.1)	2.2<br>(0.7)	94.6<br>(0.8)	92.4<br>(01.1)	2.2<br>(0.6)
